# The Confrustion Constellation: A New Way of Looking at Confusion and Frustration

**DOI:** 10.1111/cogs.70035

**Published:** 2025-01-21

**Authors:** Ryan S. Baker, Elizabeth Cloude, Juliana M. A. L. Andres, Zhanlan Wei

**Affiliations:** ^1^ Department of Learning, Teaching, and Literacies University of Pennsylvania; ^2^ Faculty of Education and Culture Tampere University

**Keywords:** Affect in learning, Emotion models, Emotion theory, Confusion, Frustration, Confrustion

## Abstract

There has been considerable research on confusion and frustration that has treated them as two unitary constructs, distinct from each other. In this article, we argue that there is instead a constellation of different types of confusion and frustration, with different antecedents, manifestations, and impacts, and that the commonalities between many types of confusion and frustration justify thinking of them as part of the same constellation of affect, distinct from other prominent affective categories. We discuss how these types of affect have been considered historically and in key models. We then discuss unusual manifestations of each form of affect that have been documented in the literature, and what light they shed on the broader constructs. We conclude with a discussion of a new theoretical framing that treats confusion and frustration as a confrustion constellation, and the opportunities and open questions that this perspective presents.

## 1 Introduction

The important role that affective states play in learning processes has been long‐known (Linnenbrink, 2006) and has been an area of focus for researchers over the last two decades. Within that focus, there has been about a decade of intensive study on the affective experiences of confusion and frustration during learning (e.g., D'Mello & Graesser, 2014; Liu, Pataranutaporn, Ocumpaugh, & Baker, 2013).

Confusion has been generally defined as the affective experience that occurs around experiences of *cognitive disequilibrium* (Piaget, 1952) or *cognitive dissonance* (Festinger, 1957), where individuals encounter incongruence with their current knowledge, and as a result, struggle to comprehend or adapt to these impasses. Although there is debate and some degree of inconsistency in exactly how confusion is defined (e.g., D'Mello & Graesser, 2014; Rozin & Cohen, 2003), what remains consistent is the understanding that experiences of confusion rely on a *mismatch of information* (D'Mello & Graesser, 2014) between what a learner knows and the information they encounter.

Frustration has been defined as an individual's experience when they are hindered from attaining a goal. Often, this experience of encountering impasses or failing to accomplish goals can lead to feelings of irritation or lack of confidence. The frustration process has been classically conceptualized as involving a frustrating situation in the presence of goals and expectations, a change in tension, and a range of common reactions to that frustrating situation (Britt & Janus, 1940). Some theories around frustration conceptualize it as the response to periods of arousal that are inhibited, resulting in the suppression of cognitive activity (Yulis San Juan & Murai, 2021). Additionally, Pekrun and Stephens (2012) have argued that when frustration occurs in an educational setting, it can be experienced as either an epistemic or an achievement emotion, depending on where attention is directed. When cognitive incongruity is emphasized, frustration arising from a student's inability to solve a problem can be seen as an epistemic emotion. However, if the focus is on students’ learning outcomes, such as failing or succeeding on an exam, then the resulting frustration is classified as an achievement emotion (Pekrun & Stephens, 2012).

A recent systematic review of studies examining the relationship between affect and learning (Karumbaiah, Baker, Tao, & Liu, 2022) found that confusion and frustration were among the most widely studied affective states in relation to learning, second only to engaged concentration/flow. Across the 39 studies they considered, they found that there was a complex relationship between confused and frustrated states and learning outcomes. Overall, Karumbaiah et al. (2022) found that the relationship between affect and learning depended on contextual variables such as methods of measurement, phases of learning during which the affect was observed, and demographic variables.

In a similar vein, studies also show that the details of the affective experience of confusion and frustration matter considerably for how these affective states impact the learning outcomes associated with them. For example, Lee, Rodrigo, Baker, Sugay, and Coronel (2011) found that learners who experienced prolonged periods of confusion had poorer learning outcomes, possibly indicating that the confused state was left unresolved. When confusion was resolved, however, learner outcomes were more positively associated with achievement scores (Lee et al., 2011). This is in line with other research finding that the resolution of confusion impacts the experiences of learning. Experiences of confusion encourage knowledge exploration after the submission of incorrect answers (Vogl, Pekrun, Murayama, Loderer, & Schubert, 2019) and once confusion is resolved, a student is able to transition back into positive affective states (Di Leo, Muis, Singh, & Psaradellis, 2019). However, if a student is unable to overcome the source of their confusion, this state will persist or will contribute to experiences of frustration that, in turn, may transition into anger or hopelessness that are detrimental to their learning outcomes (Di Leo et al., 2019).

At the same time, several commonalities can be noted between confusion and frustration, both in terms of their causes and their impacts on learning and achievement. For instance, both stem from an inability to understand or solve a problem, where confusion is characterized by uncertainty about the cause (Lodge, Kennedy, Lockyer, Arguel, & Pachman, 2018), while frustration is characterized by an inability to overcome an obstacle or achieve a goal (Alon & Nachmias, 2020; Dollard, Miller, Doob, Mowrer, & Sears, 1939). Perhaps because of this, both confusion and frustration are often defined in terms of an impasse that is reached—where the individual has reached some factor that is blocking their progress in some fashion, and is unable to immediately resolve that impasse (Lodge et al., 2018). Additionally, both frustration and confusion can trigger social functioning behaviors during the learning process, such as seeking assistance from instructors or peers. These two affective states also both influence students’ utilization of cognitive and metacognitive learning strategies, and can lead to the same strategies such as help‐seeking (Rowe & Fitness, 2018). The research on confusion and frustration has often treated these two affective states as separate and distinct, but these commonalities call this separation into question. Also, some of the substates of each of these affective states appear more similar to each other (duration, learning impact) than to their states’ standard manifestations. Take duration, for example: the relationships discussed above between brief confusion and learning are similar to the relationships between brief frustration and learning, while the relationships between extended confusion and learning are similar to the relationships between extended frustration and learning (Liu et al., 2013).

Building on the shared characteristics between confusion and frustration highlighted above, we consider an alternate framing within this paper—treating confusion and frustration as part of a *confrustion constellation*. Some papers have already used a measurement approach that combines confusion and frustration (Mogessie, Richey, McLaren, Andres‐Bray, & Baker, 2020; Richey et al., 2019; Richey et al., 2021). We discuss the literature around these two constructs, considering evidence of commonalities and differences, and evidence that these two constructs themselves incorporate a range of subconstructs. In short, the current focus of most of the field treats confusion and frustration as two unitary constructs, distinct from each other. We argue that instead, there is a constellation of different types of confusion and frustration, with different antecedents, manifestations, and impacts, and that the commonalities between many types of confusion and frustration justify thinking of them as part of the same constellation of affect (meaningfully distinct from, say, boredom or engaged concentration/flow). We select the term constellation to refer to our theoretical model, to illustrate the idea that while confusion and frustration manifest in a number of ways, there are meaningful commonalities among all of the manifestations.

### 1.1 Models of emotions/affect

The current standard conceptualization of confusion and frustration as unitary, distinct states emerged from specific models of affect or emotion. While that usage aligns most closely within the “affective states”/“academic emotions”/“epistemic emotions” frameworks seen in the writing of authors such as Pekrun (2006) and Graesser (2011), the same set of emotion/affect constructs can be seen in a broader range of frameworks, most of which represent affective states as distinct and unitary—with key exceptions such as the valence/arousal framework seen in Russell, Lewicka, and Niit (1989). In this section, we briefly review some of the key models of emotions/affect and how confusion and frustration are viewed within these models.

Before doing so, it may be worth briefly considering what “affect” and “emotion” are. Affect has been defined as an individual's experiential state that can be influenced and can influence their neurobiology, psychophysiology, and consciousness (e.g., D'Mello, Blanchard, Baker, Ocumpaugh, & Brawner, 2014; Izard, 2010; Picard, 1997). Emotions often occur in response to environmental stimuli, and are experienced in brief episodes that can last between a few seconds and minutes, during which they can be expressed through physical motion (Morita, Nagai, & Moritsu, 2013). These experiences are intense and explicit manifestations of physiological and behavioral responses that are “at the forefront of consciousness” (D'Mello et al., 2014; Ekman, 1984; Ekman, 1994). In practice, in many research communities, the terms “affect” and “emotion” are used interchangeably, with different papers (even by the same authors) using each of these terms as the higher‐level category for the same lower‐level constructs (such as confusion and frustration).

#### 1.1.1 The circumplex model

**Fig. 1:**
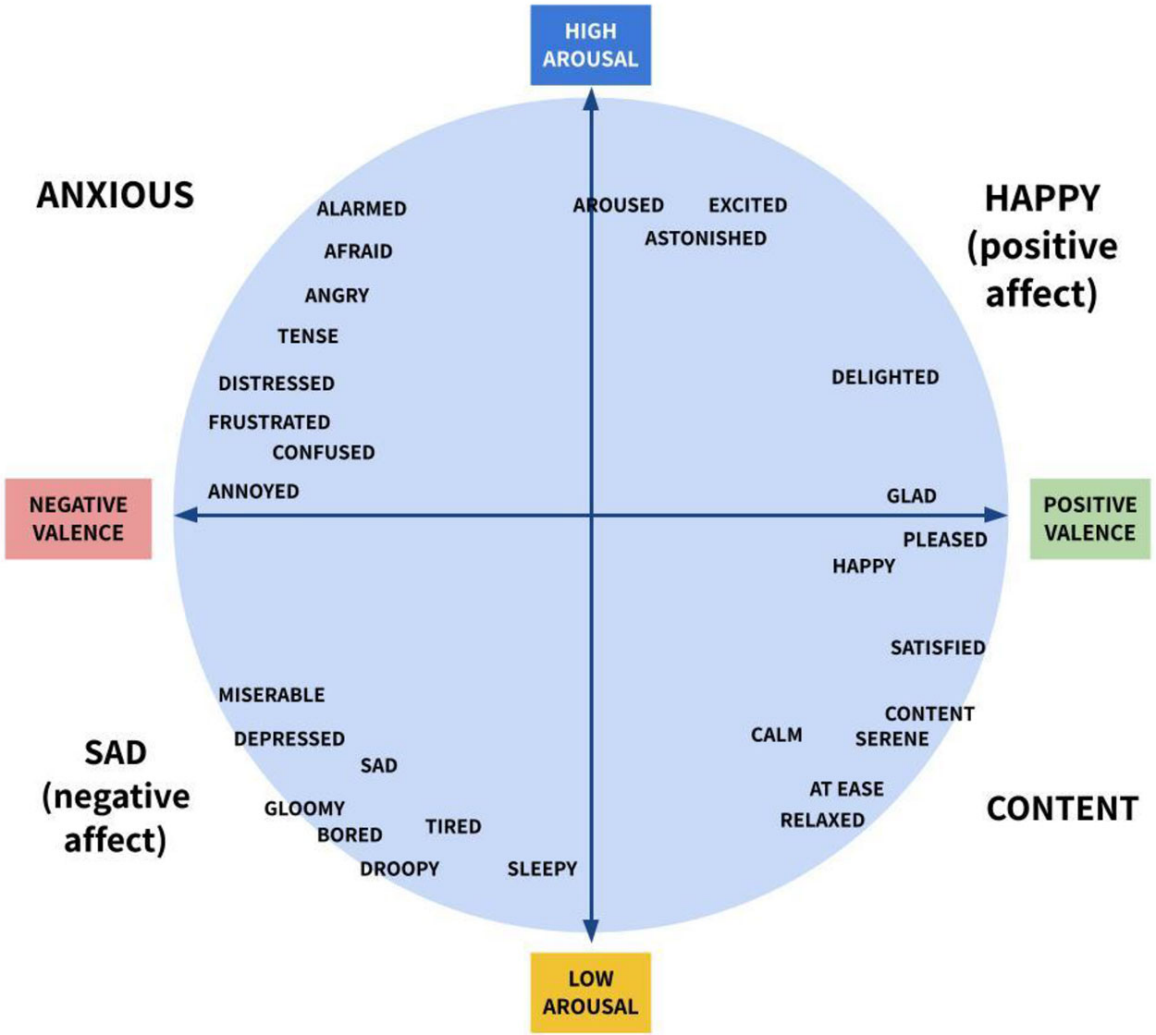
Circumplex representation of feelings shown in its two‐dimensional scaling (adapted from Liu et al., 2018; Plutchik & Kellerman, 2013; Russell et al., 1989; Sharar et al., 2016; Zagalo et al., 2005; and Dillon et al., 2016).

One of the simplest models of affect, the circumplex model (Russell et al., 1989), depicts emotions as distributed in a circular space based on two primary constructs: valence (the pleasure‐displeasure continuum) and arousal (the activation‐deactivation continuum) (Colibazzi et al., 2010; Gerber et al., 2008; Posner, Russell, & Peterson, 2005; Tseng et al., 2014) based on evidence for both valence and arousal in early neuropsychological models. The circumplex model does not provide clear distinctions between emotions; instead, it argues that emotions blend into another through “imperceptible gradations” (McDougall, 2015, p. 114; Russell & Fehr, 1994). The only definite boundaries the circumplex model provides are presented in its four quadrants: (1) happy, characterized by positive valence and high arousal; (2) anxious, characterized by a negative valence and high arousal; (3) sad, characterized by a negative valence and low arousal (also called depression); and (4) content/relaxation, characterized by a positive valence with low arousal. However, finer divisions can be made to suit specific purposes (Plutchik & Kellerman, 2013). As Fig. 1, drawn from multiple representations of the circumplex model (i.e., Plutchik & Kellerman, 2013; Liu et al., 2018; Russell et al., 1989; Sharar et al., 2016; Zagalo, Torres, & Branco, 2005, and Dillon et al., 2016), shows, both confusion and frustration are seen (relatively close to each other), within the anxious quadrant. The exact placement of confusion and frustration in this model comes from a combination of Sharar et al. (2016), which places frustration as highly negative valence and above medium arousal, and Dillon et al. (2016), which places confusion below frustration and to its right. (Frustration is, however, placed low in relative arousal compared to Dillon et al. (2016), as that paper did not consider higher arousal states such as angry or afraid.) This model, while representing the relative closeness between confusion and frustration in terms of these two dimensions, does not capture other aspects of their relationship.

#### 1.1.2 Basic emotions

The theory of basic emotions (Ekman, 1992) is one of the most widely cited theories of emotion. This theory argues that there are only six basic and universal emotions: anger, disgust, fear, happiness, sadness, and surprise. According to Ekman's theory, these six emotions remain constant across divisions of culture and people, typically defined based on certain combinations of features such as (1) the speed of onset of the affective experience, (2) duration, (3) automatic appraisal, and (4) coherence of responses (Ekman, 1992). The foundational assumptions of this theory are that these six emotions exist and that they are distinct from one another, emerging due to evolution in order to “mobilize an organism to deal quickly with important interpersonal encounters” (Ekman, 1992, p. 171).

However, each of these six basic emotions refers to a cluster of related states, or “emotion families” (Ekman, 1992). Each of these *emotion families* contain subemotions that are independently characterized from one another within social contexts and are highly influenced by environmental cues, individual differences, biological thresholds for reactivity, and variations in learning experiences (Ekman, 1992). For example, the basic emotion of anger can manifest as guilt, frustration, or dislike (Roseman, 1994), which are distinct from each other but are also related in ways that none are related to emotions from a different emotion family (Ekman, 1992).

Within this theory, emotions within the anger family involve a tendency to move away from or against something. In the case of frustration, an individual responds against inanimate objects or events in ways that correspond to direct “forceful action” against the source of frustration (Amsel, 1992; Roseman, 1994). Within Ekman's (1994) theory, frustration can also be viewed as a condition that contributes to anger, as the theory treats “emotion labels [as] a shorthand that refers to any of a number of different aspects of an emotion—including antecedents, expressions, memories, and consequences” (Ekman, 1994, p. 275).

In contrast to frustration, to the best of our knowledge, confusion is never explicitly mentioned as an emotion within Ekman's writings on basic emotions.

#### 1.1.3 Epistemic emotions

In the last decade, an alternate framework has emerged as the culmination of multiple research initiatives across different research teams, which represents emotion in terms of specific “epistemic emotions” (D'Mello & Graesser, 2012; Nerantzaki, Efklides, & Metallidou, 2021). The term “epistemic emotions” refers to the fact that these emotions emerge from “epistemic, knowledge‐generating cognitive activities” (Pekrun & Linnenbrink‐Garcia, 2014, p. 4). The terms “academic emotion” and “achievement emotion” are also used, as these emotions directly involve academic and achievement‐related activities (e.g., studying) and outcomes (e.g., success and failure) (Pekrun, 2006; Pekrun & Perry, 2014). The term “affective states” is probably used even more frequently for these emotions than either of these terms, in the broader educational literature (e.g., D'Mello et al., 2014), stemming from key early work by Picard (1997).

One of the first sets of emotions/affective states of this nature was proposed by Craig, Graesser, Sullins, and Gholson (2004) and included frustration, confusion, boredom, flow (or engaged concentration), and eureka. The selection of these five affective states was guided by a model from Stein and Levine (1990) which identified a theoretical link between a person's goal and their emotional disposition. According to this model, individuals’ preferences for certain states are influenced by their interpretation and accommodation of external stimuli (Stein & Levine, 1990).

Epistemic emotions manifest in response to events (Craig et al., 2004) and confusion and frustration are common such responses, along with boredom and flow/engaged concentration (Craig et al., 2004; Karumbaiah et al., 2022). Work by D'Mello et al. (2005) suggested that when the amount of changes in the environment and stimuli are high, curiosity and surprise may be triggered; when the potential for goal achievement is low (or blocked), anger, frustration, sadness, or fear may be triggered; and when both the changes in the environment and potential for goal achievement are high, happiness, joy, and eureka may be triggered. Based on this general paradigm, the control‐value theory of academic emotions has argued that the occurrence of epistemic emotions (academic emotions) is based on appraisals of control and value (Pekrun, 2006).

This breakdown is reminiscent of another popular emotion model, OCC (Ortony, Clore, & Collins, 1988), which is not discussed in detail here due to not including or considering confusion or frustration. OCC uses a typology that identifies three main points of focus that an individual follows in response to various stimuli. These three points of focus are (1) desirability of events, (2) actions of agents, and (3) appeal of objects (Ahmadpour, 2014; Ortony et al., 1988; Ortony, Clore, & Collins, 2022). Considering the cognitive factors that lead to different emotions may be a useful step for theoretical models that attempt to understand specific emotions in greater detail.

## 2 Confusion and frustration

### 2.1 Early understanding of the two emotions within the context of learning

In the late 19th and early 20th centuries, frustration was widely accepted as a natural byproduct of learning, but confusion during learning was considerably less emphasized. Much of this literature originated from the psychology of animal learning and motivation. Work toward developing theory on frustration in this era adopted a stimulus‐response paradigm (e.g., Thorndike, 1898). This framework explains the role of anticipation, expectancy, and memory during learning framed as classical conditioning. In response, Amsel (1958) developed a frustration theory anchored in classical conditioning (Pavlovian) and dispositional learning (Thorndikian). These paradigms explain basic learning processes that are linked to basic extrinsic rewards, driven by motivation systems that create reward‐schedule effects (Amsel, 1994).

Within the learning frameworks used in these theories, reward schedules—sequences of rewards and nonrewards—involve three types of events: (1) rewarding, (2) punishing, or (3) frustrative, the absence or delay of a rewarding event in a situation where it had been previously provided. Several types of frustration are defined (Rosenzweig, 1944). First, primary frustration is a buildup of situations in which there is already a conditioned anticipatory response, but the response is not rewarded. This is known as the frustration effect and it stems from the deprivation of reward when an unconditioned response of frustration occurs due to stimulus‐nonreward pairs and anticipatory responses. In contrast, secondary frustration stems from a conditioned, or learned, response to obstacles in satisfying a need or meeting a goal. This type of anticipatory frustration is learned from the conditioning of stimulus‐nonreward pairs, and it is hypothesized that this type of frustration increases in strength as a function of nonrewarded trials (Amsel, 1958; Amsel, 1962). Overall, this model of frustration theory describes multiple properties of frustrated states, which depend on conditioned expectancies and their failed confirmation. Specifically, Amsel (1994) argued that these diverse properties of frustration can have energizing, directive, and suppressive effects on learning and memory.

Within this model, the extent to which frustration demonstrates one of these effects over another depends on the barrier preventing the learner from attaining the goal‐related reward. For example, obstacles that prevent a student from meeting a learning goal can be appraised as either external or internal. A student might appraise an obstacle as external based on the resources they have access to in the classroom, for instance. If the instructor is busy with another student, they may perceive this as an external obstacle toward meeting a goal when their question is left unanswered. In contrast, a student may judge an obstacle as internal if they perceive they cannot make progress based on their own weaknesses or lack of capability. Interestingly, while increased motivation tends to be associated with the frustration stemming from internal factors such as an inability to grasp a concept or dissatisfaction with one's current level of knowledge, frustration toward external factors such as busy instructors appears to be more harmful to learners’ motivation (Rowe & Fitness, 2018). Frequent frustrated events can increase the strength of a later frustrated state and lead to behaviors such as aggression (Dollard et al., 1939). This effect has long been studied in animal studies, where frequent and prolonged frustration events predict energizing properties in later frustration events (Berkowitz, 1989). By contrast, if frustration is experienced less often, the frustration effects are not as energizing or aggressive. Similarly, in school settings, researchers found that the frequent frustration experienced by students—associated with material the student does not want to study, constant high expectations from teachers and parents, or strict disciplinary rules or punishments—often induces aggressive behaviors, particularly among secondary school boys (Fatima & Malik, 2015). Some of these differences manifest in other ways; for example, one study examined the frequency and recurrence of facial expressions that co‐occurred with frustration and confusion (Cloude et al., 2024). They discovered that frustration often was associated with different facial expressions (e.g., disgust and surprise) for different students; a higher occurrence of disgust facial expressions during frustration was linked to less prior knowledge, and when disgust was repeatedly expressed during frustration, it was associated with lower learning gains.

More generally, research has found inconsistent relationships between frustration and learning‐related behaviors and outcomes. For example, in the work of Lopatto et al. (2020), observations collected from over a hundred faculties and thousands of students revealed that frustration can play a critical *positive* role in fostering students’ development and growth. Many faculty members reported that intellectual challenges or even struggles (i.e., computer problems, time challenges, group dynamics) can positively impact students’ learning; as these obstacles are conquered, students can achieve a sense of accomplishment. In other work, however, frustration has been found to be associated with students avoiding challenging tasks, abandoning their learning efforts, or simply learning less (D'Mello & Graesser, 2012).

Research on confusion during learning was initially shaped by developments in the cognitive and psychological sciences, to explain cognitive dissonance and a lack of understanding during a learning event. However, confusion is viewed in very different ways in different models and accounts. For example, some modern theorists consider confusion a basic emotion (Cowen & Keltner, 2021), while others argue that confusion is a state based on cognition (Clore, 1992). In other work, based on attribution theory, confusion is defined as an affective state, and the cognitive component is based on its appraisal structure (Lazarus, 1991; Scherer, 2001). In this view, the process of confusion is based on a cognitive interpretation, following feelings or an emotional response related to the experience of a lack of understanding during information processing.

A key assumption of this perspective is that for confusion to occur in learning, the learner needs to be attempting to learn or understand something. Confusion may result from the interpretation of feelings observed, due to the learner monitoring their cognitive processing in relation to their learning goals, values, knowledge, and/or abilities (Silvia, 2010). Going further, Silvia (2010) argues that confusion stems from an appraisal of feelings of high novelty and low comprehensibility of information being cognitively processed. Ellsworth (2003) also explained that confusion may stem from feelings of uncertainty. This work connects to earlier work by Berlyne (1960), which outlined several components that contribute to feelings of uncertainty, including the degree of novelty, complexity, conflict, and unfamiliarity during information processing. Interestingly, this perspective in turn originates from early emotion theories proposed by Dewey (1895) and Hebb (1949), which argue that conflict invokes perception and an emotional response. Based on this, Berlyne claimed that confusion and primary frustration are connected by similar types of conflict—there is an anticipatory expectation to achieve a goal, yet there is a block or conflict in meeting this goal that results from an external source. However, secondary frustration is distinguishable from confusion and primary frustration, as the source of conflict involves a conditioned response about the goal barrier resulting from an internal source.

Berlyne (1960) argued that confusion may stem from information processing that evokes multiple concepts, resulting in a cognitive conflict. However, Berlyne notes that not all conflicts are the same, and hypothesizes that the type and severity of the conflict may elicit varying degrees of confusion or primary frustration. Similarly, Lodge et al. (2018) suggest that feelings of difficulty that may arise from conflict likely impact the elicitation of confusion as well, causing some forms of confusion to be more productive, while other forms are more unproductive for learning. The combination of how challenging an activity or task is, and a student's level of knowledge/skill, may determine whether confusion falls within the zone of proximal confusion (ZPC) (Graesser, 2011). The ZPC framing explains that there is a threshold where productive confusion becomes nonproductive. First, a student encounters disequilibrium based on an impasse in the learning process. The student stays within the state of productive confusion as long as they stay sufficiently engaged and appraise the impasse as being confusing. When this occurs in a productive way, the student relies on metacognitive awareness and skill to monitor and recognize that confused feelings are a cue to adapt their strategy. They expect that the disequilibrium can be resolved, conceptual change will occur, and they can make progress toward their learning goal. However, if confusion becomes persistent, the student may possibly move into the zone of suboptimal confusion. This is when confusion becomes unproductive and can lead to frustration and/or boredom (Lodge et al., 2018).

Just as there are several factors that impact the emergence and effects of confusion, individual differences also impact if, how, and when confusion emerges (Kennedy & Lodge, 2016). In particular, within this account, the threshold that determines when confusion is productive or unproductive is defined by the individual's degree of tolerance of possibly negative emotions. In other words, the threshold may depend on how well the student tolerates and can adapt to feelings of novelty, uncertainty, and difficulty, in relation to the goals/drive they have to persist through the task.

This rich history of studying emotions and their underlying mechanisms has paved the way for a comprehensive examination of confusion and frustration. Initially treated as distinct emotional experiences, researchers have begun to uncover the intricate connections between these two states and their impact on cognitive processes. Over time, accounts have suggested that confusion and frustration may be highly linked, where confusion and frustration are connected on a continuum of cognitive equilibrium (e.g., D'Mello & Graesser, 2012), where their effects can either benefit or disrupt learning.

### 2.2 Confusion and frustration as discrete emotions

A considerable amount of research has been conducted on confusion and frustration during learning. This work, which has treated confusion and frustration as discrete, static (negatively valenced) categories, has found important links between confusion, frustration, and learning outcomes (Karumbaiah et al., 2022; Lodge et al., 2018). Across these studies, confusion and frustration are differentiated in a variety of ways: using self‐report data, classroom observations, video recordings, verbal protocols, and physiological signals (AlZoubi, D'Mello, & Calvo, 2012; Kapoor, Burleson, & Picard, 2007). One of the most common methods involves collecting self‐reported confusion and/or frustration before, during, or after learning using validated instruments (e.g., Epistemically Related Emotions Scale [Pekrun, Vogl, Muis, & Sinatra, 2017]).

Self‐report data capture the learner's subjective judgment of their own confusion and/or frustration (Pekrun et al., 2017). This method is often used because of the importance of the learner's interpretation to the manifestation of epistemic emotions (Sabourin, Mott, & Lester, 2011; Schachter & Singer, 1962), where the individual learner determines the meaning of an affective experience and this interpretation determines how the subsequent emotion is experienced. Other approaches involve either quantitative field observations in formal learning settings (e.g., BROMP [Ocumpaugh, Baker, & Rodrigo, 2015]), retrospective interviews of affective judgments (D'Mello, Craig, Sullins, & Graesser, 2006; McDaniel et al., 2007), or verbal protocols such as emote‐alouds, where learners verbalize their affective states in‐situ (Di Leo et al., 2019). Other studies involve a combination of these methods, such as the combination of BROMP and self‐report in Baker, D'Mello, Rodrigo, and Graesser (2010).

In addition, automated algorithms can ubiquitously collect confusion and frustration in‐situ at granular and continuous rates over time (D'Mello & Graesser, 2014). These automated “detectors” typically recognize affect using either logs of how a learner interacts with materials in a digital learning environment (Botelho, Baker, & Heffernan, 2017; Jiang et al., 2018), or facial cues via facial tracking systems and facial expression recognition models (Cloude et al., 2022; Dever, Wiedbusch, Cloude, Lester, & Azevedo, 2022; Kapoor et al., 2007; Suresh et al., 2021), although other sensors have also been investigated, including eye tracking (Lim, Mountstephens, & Teo, 2020) and physiology (e.g., galvanic skin responses and pressure applied by the learner's hand to the computer mouse) (Kapoor et al., 2007). These automated detectors have been used for a number of purposes: to investigate the learning impacts of confusion and frustration (see review in Karumbaiah et al., 2022), to investigate how children and adults reason about others’ emotions (Ong, Asaba, & Gweon, 2016), to study longer‐term impacts of affect during learning (Almeda & Baker, 2020; San Pedro, Baker, Bowers, & Heffernan, 2013), to study how affect shifts over time (Botelho, Baker, Ocumpaugh, & Heffernan, 2018; D'Mello & Graesser, 2012; D'Mello, Taylor, & Graesser, 2007), to study how affective feedback influences learning (Hayashi, Matsumoto, & Ogawa, 2012), and to study how affect varies between populations (Karumbaiah, Ocumpaugh, & Baker, 2022), among other applications.

Within this paradigm, however, several studies have raised questions about the degree to which confusion and frustration are generally separate. As previously mentioned, researchers receiving training on distinguishing affective states have often struggled to distinguish confusion and frustration (Ocumpaugh et al., 2015). Bosch and D'Mello (2014) also found an overlap in confusion and frustration using retrospective affective judgment protocols, finding that confusion and frustration co‐occurred above rates of chance. Later on, Bosch and D'Mello (2017) found that confusion and frustration were one of the most frequent co‐occurring pairs of emotions, and that confusion and frustration demonstrated reciprocal relationships: learners frequently transitioned from confusion to frustration, and from frustration to confusion. Similarly, Di Leo et al. (2019) also identified frequent co‐occurrence in student verbal utterances about confusion and frustration, such as “Ugh, this is taking forever! [frustration]. Like I said before, I'm confused [confusion].” In line with these studies, Dillon et al. (2016) found that confusion and frustration were more likely to co‐occur in self‐reports than any of 91 other possible emotion combinations analyzed, and that the co‐occurrence rate was significantly above what would be expected by chance, replicating the finding seen in Bosch and D'Mello (2014).

Liu et al. (2013) utilized both field observations and text replays (human coding of interaction log data) to measure confusion and frustration during learning. They examined confusion and frustration both independently and in combination to assess impacts on learning outcomes. Their results showed that sequences of confusion and frustration were beneficial for learning, but only when they both occurred in brief periods of time. In contrast, learning was negatively impacted when sequences of confusion and frustration persisted for longer periods of time. Moreover, this effect was the most pronounced when confused and frustrated sequences were analyzed together, rather than independently. In sum, these findings suggest that confusion and frustration demonstrate similar properties and patterns when they are experienced during learning.

However, not all studies found these consistencies. Botelho et al. (2018) found that confusion transitioned to frustration, but frustration did not transition into confusion, using data from affect detectors built using deep learning models to detect instances of confusion and frustration within interaction logs (the models were developed using classroom observation data). However, they still found that confusion and frustration demonstrated similar rates of decay—the probability of the affective state persisting for a specific duration of time. More broadly, a meta‐analysis by Karumbaiah, Baker, Ocumpaugh, and Andres (2023) found that confused students were less likely rather than more likely to become frustrated, and that frustrated students were neither more nor less likely to become confused. Cloude et al. (2022) found a negative interaction between facially expressed confusion and pre‐test scores on scientific reasoning, but not for frustration.

### 2.3 Confusion and frustration treated as unitary

Based on the results of studies finding similar patterns for frustration and confusion (though not a consistent pattern of transitions between them), other work has treated confusion and frustration as a single combined emotion (Mogessie et al., 2020; Richey et al., 2019; Richey et al., 2021). This work has used the term *confrustion* to refer to the merger of these two affective states, and has used human labels of confrustion (confusion and/or frustration) from interaction log data, as well as automated detectors built using these human labels.

This body of work started by attempting to replicate work by (Lee et al., 2011) that identified confusion using text replays (human coding of interaction log data displayed in an easy‐to‐read form) but researchers found it highly challenging to differentiate confusion from frustration solely from viewing log data. The researchers noted that this challenge was not unique to text replays; classroom observers also often have high rates of disagreement in terms of these two affective states (Liu et al., 2013). Based on this, and their past classroom observation experience where the interaction behaviors observed for the two affective states had been similar, the researchers decided to consider confusion and frustration together—acknowledging that they could not distinguish which of these a student was experiencing—and termed the construct they were measuring *confrustion*.

This methodological choice, potentially risky (if two very different constructs were being combined), nonetheless led to many statistically significant findings. For example, Richey et al. (2019) built confrustion detectors using interaction log data and labels generated using text replays—using machine learning to identify behaviors within the interaction log data (such as delays in specific circumstances or patterns of errors over time) that matched the human‐created labels. They then compared the rate of confrustion between two experimental conditions: erroneous examples and problem solving with a math tutor. The erroneous examples were designed with misconceptions, hypothesized to promote deeper learning. They hypothesized that this would lead to better learning outcomes based on earlier work on inducing confusion by contradiction and simulated student errors (Lehman et al., 2013). Their results showed that learners in the erroneous examples condition had significantly more and longer periods of confrustion than the problem‐solving group. Surprisingly, confrustion was negatively associated with immediate and delayed knowledge assessment scores across both conditions, but learners in the erroneous examples condition performed significantly better on the delayed knowledge assessment than the problem‐solving groups. Additional analyses revealed that across both groups, learners tended to demonstrate less confrustion later than earlier in the learning session, but this varied across the course of the intervention.

A follow‐up study by the same group found that confrustion was associated with a lower frequency of gaming the system behavior (a type of behavioral disengagement) (Mogessie et al., 2020). Another follow‐up study by the group compared confrustion and learning between two experimental conditions: a digital math learning game and an intelligent tutor (Richey et al., 2021). Their results showed that learners in the game condition showed significantly more confrustion, while the nongame condition demonstrated significantly more gaming of the system. Confrustion had no impact on learning outcomes in the game condition, but the proportion of confrustion was associated with better learning outcomes in the nongame condition.

Overall, then, treating confusion and frustration as a single construct of *confrustion* still led, perhaps surprisingly, to a workable measure that correlated to other constructs.

### 2.4 Confusion and frustration: Is either even unitary?

As the previous section shows, merging confusion and frustration does not appear to lead to an incoherent construct that does not correlate to anything. Instead, the merged construct appears to have explanatory power. This raises the possibility that the two affective states may blend into each other to some degree, at least in some contexts. Another relevant question is, are there meaningful divisions *within* these affective states? (Possibly cutting across the two states.)

We first consider the impact of whether confusion and frustration are resolved or not. In response to early findings that the relationship between confusion and learning was inconsistent, Lehman, D'Mello, and Graesser (2012) studied the conditions under which confusion could be positive for learning. They found that confusion, when resolved (even just partially), was associated with better learning outcomes. By contrast, if confusion was not resolved, poorer learning outcomes were obtained. Furthermore, they demonstrated that inducing confusion through the design of activities could improve learning outcomes, a finding replicated in their later work (Lehman & Graesser, 2015; Lehman et al., 2013). They hypothesized that if learners could learn to regulate their learning strategies better in response to confusion, they would learn to learn better. However, Zhang et al. (2021) found in a very different learning system that although confusion prompted the greater use of meta‐cognitive strategies, those strategies did not lead to better learning and did not resolve the confusion.

However, this line of research did not consider whether the same patterns held for frustration as confusion. As previously discussed, there is some reason to think that frustration and confusion might behave in similar ways in terms of this phenomenon, as seen in the study by Liu et al. (2013) discussed earlier, where confusion and frustration had similar relationships to other measures and could even be used interchangeably in analyses of the duration of the two affective states and post‐test scores.

Confusion and frustration, therefore, seem to correlate differently to learning depending on whether they are brief/resolved or extended/unresolved. It is unclear whether these two types of confusion and frustration (brief or extended) are experientially, cognitively, and phenomenologically the same, or if they are distinct. Are they separated solely by successful resolution—that is, by an experience external to the affective experience? Or is there a deeper difference between these two forms of confusion and frustration?

Another line of thought on this type of affect sheds light on a possibility. Take the experience that Gee (2003) refers to as pleasant frustration—a type of frustration which involves the “feeling of being highly challenging, but ultimately doable” (Gee, 2003, p. 25). Gee argues that this affective experience is particularly prominent in well‐designed games, and that part of the conditions that support its emergence is the ability to see progress toward a concrete goal. This affective state, as described by Gee, seems to have an appraisal dimension (also see Clore & Ortony, 2008), a perception of whether the task is achievable. This view on frustration is also aligned with Amsel's (1994) and Dollard et al.’s (1939) earlier work on frustration, which suggests that properties of frustration can vary, and in some cases, frustration can be energizing and directive based on anticipatory expectations, providing cues to a learner during a learning task. As such, pleasant frustration relates to the idea of brief or resolved confusion and frustration. Where brief/resolved confusion ultimately is resolved by the learner, pleasant frustration is anticipated to be resolved. Is it, therefore, possible that brief/resolved confusion and frustration may involve the same anticipation of success? And is it pleasant every time an individual experiences confusion or frustration that they expect they can resolve? Or is it something about the design of games that goes beyond the anticipation of eventual success, to create pleasure?

In summary, while there may be reason to doubt that confusion and frustration are truly separate, there also may be reason to think that one or more other dimensions—temporality, eventual success, progress toward success, the anticipation of success, and even pleasure—may differentiate types of confusion and frustration (Table 1).

**Table 1:** Various methods and operationalizations of confusion, frustration, and confrustion

Confusion	Frustration	Confrustion	Studies	Methods	Operationalization
X	X		Pekrun et al., 2017; Sabourin et al., 2011; Kapoor et al., 2007; Muis, Psaradellis, Lajoie, Di Leo, and Chevrier, 2015; Baker et al., 2010; Dillon et al., 2016	Validated self‐report instruments	Subjective judgment of feelings, either prospectively, concurrently, or retrospectively.
	X		Alon and Nachmias (2020)		
X	X		Karumbaiah et al., 2022; Baker et al., 2010	Quantitative field observations	The Baker Rodrigo Ocumpaugh Monitoring Protocol (BROMP) uses a combination of facial, verbal, and behavioral cues. Frustration is classified as a learner “presents feelings of distress or annoyance” (Ocumpaugh et al., 2015, pp. 37). Confusion is classified as a learner who “looks like they are having difficulty understanding the class materials or whatever they are most prominently engaged with” (Ocumpaugh et al., 2015, pp. 36).
X			Zhang et al. (2021)		
X	X		Liu et al., 2013; Karumbaiah et al., 2022; Botelho et al., 2017; Jiang et al., 2018; Almeda and Baker, 2020; Botelho et al., 2018; Cloude et al., 2024	Automated detection of learner−system interactions built using observation data	Features are distilled from interaction data and synchronized with external labels of confusion and/or frustration. Interaction data are parsed into “clips” of actions (typically 20 s long) that precede and co‐occur with external labels (e.g., number of help requests).
X			San Pedro et al. (2013)		
X			Lee et al. (2011)	Human coding of learner−system interactions “Text replays”	Features are distilled from interaction data into “clips” of actions (typically 20 s long) and displayed trained coders identify affective states from sequences of learner interactions over time.
		X	Richey et al., 2019; Mogessie et al., 2020; Richey et al., 2021; Richey et al., 2019		
X	X		Cloude et al., 2022; Dever et al., 2022	Facial recognition software	Facial landmarks are mapped onto emotional classification configuration defined using the Facial Action Coding System (FACS) (Ekman & Friesen, 1978). One example is a furrowed brow and tightened lips.
	X		Kapoor et al. (2007)		
	X		Kapoor et al. (2007)	Physiological signals	Physiological and physical measurements such as electrocardiogram, electromyogram, eye movements, posture, and galvanic skin response
X			AlZoubi et al. (2012)		
X	X		Di Leo et al., 2019; D'Mello et al., 2006; Craig, D'Mello, Witherspoon, and Graesser, 2008	Verbal protocols or emote‐alouds	Frustration is defined as verbal expressions of irritation or dissatisfaction. Confusion is defined as verbal expressions of being puzzled or mixed up about the learning content.
X	X		Karumbaiah et al., 2022; D'Mello and Graesser, 2012; Graesser et al., 2006; D'Mello et al., 2006; McDaniel et al., 2007; D'Mello, Chipman, and Graesser, 2007; D'Mello et al., 2007; Bosch and D'Mello, 2014; Bosch and D'Mello, 2017	Retrospective human coding	Video coding (self, peers, or trained judges using FACS) of facial expressions (typically at 20‐s intervals). Frustration was defined as “dissatisfaction or annoyance” (Graesser et al., 2006, pp. 287). Confusion was defined as “a noticeable lack of understanding” (Graesser et al., 2006, pp. 287).
X			Lehman et al., 2013; Lehman et al., 2012; Lehman and Graesser, 2015		

## 3 Reconceptualizing confusion and frustration as confrustion: Implications for theory, research, and learning design

### 3.1 The confrustion constellation

One possible way to address the complexity of findings around confusion and frustration—and the frequent commonalities in findings about these two affective states—is to stop treating them as two affective states, and start treating them as part of a constellation of affective experiences, within a multidimensional space: a *confrustion constellation*. Doing so acknowledges that there are many ways that confusion and frustration emerge and manifest, and that some forms of confusion may be more similar to some forms of frustration than to other forms of confusion.

The commonalities between frustration and confusion are clear, both empirically (as discussed above) and conceptually—individuals experience both emotions when their cognitive structures are misaligned with new input. In educational settings, confrustion represents a set of experiences that are driven by a similar underlying goal of gaining knowledge about a topic or actively engaging in the problem‐solving process, emphasized by the epistemic connections between these two states. In other words, the range of experiences involving frustration and confusion is rooted in their shared epistemic nature, as both can be defined as epistemic emotions, and often co‐occur or morph into one another during complex learning tasks. Frustration and confusion typically emerge in response to (1) an impasse or barrier preventing information processing of the topic needed for (2) achieving the learning goal of understanding the topic. Thus, these emotions are already connected by the shared goal and motivation to obtain knowledge via information processing mechanisms. More broadly, past models (e.g., D'Mello & Graesser, 2012) have noted the key role of cognitive disequilibrium—when a learner struggles to reconcile discrepancies in their understanding—in both confusion and frustration. These findings highlight the interconnectedness of confusion and frustration, suggesting that they are not merely co‐occurring affective states. Rather, they are part of a larger, dynamic process that learners continuously navigate when tackling challenging learning activities.

**Fig. 2:**
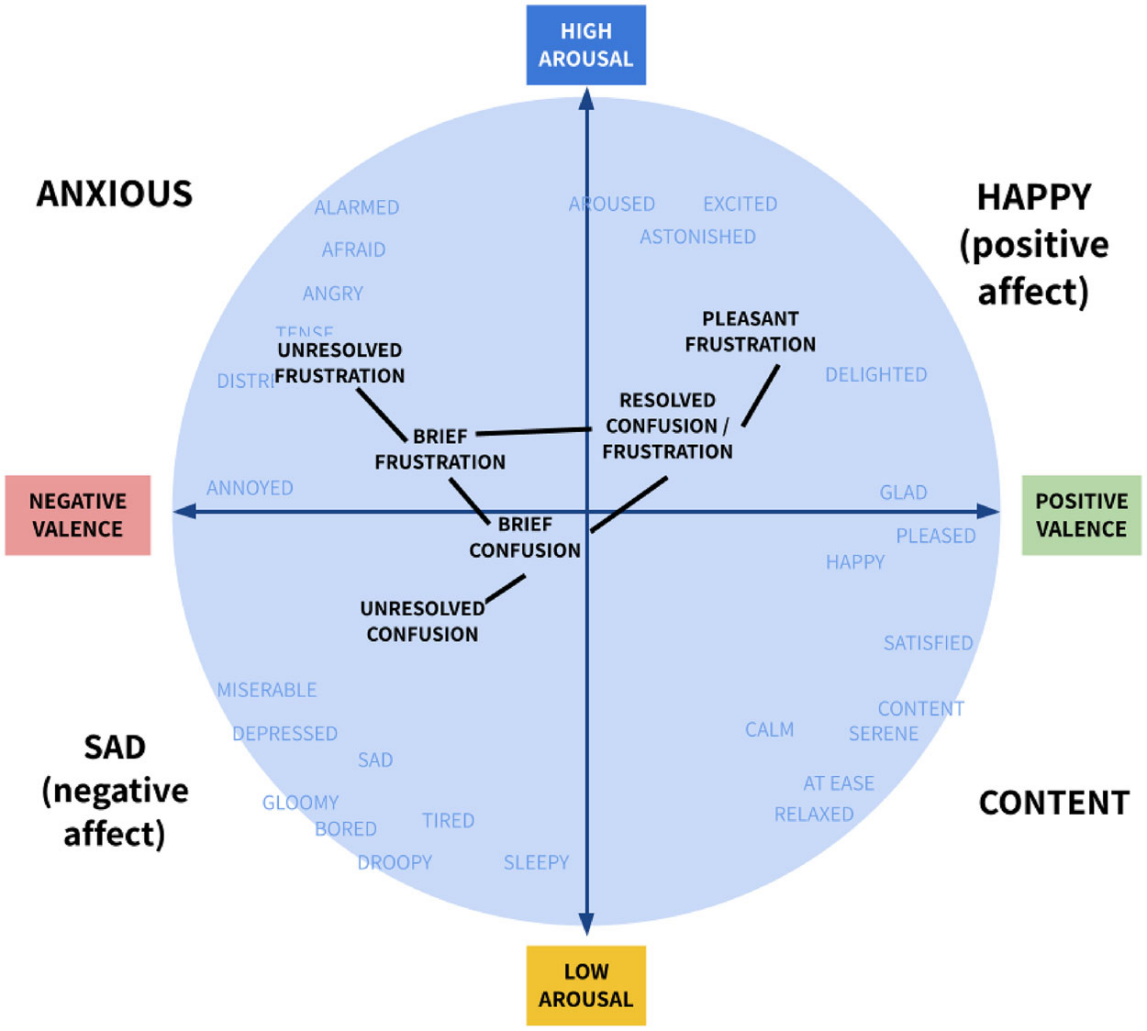
A hypothetical mapping of the Confrustion Constellation to locations within the Circumplex Model.

This constellation would include, at minimum, all of the forms of confusion and frustration discussed above: brief confusion, brief frustration, resolved confusion, resolved frustration, extended confusion, extended frustration, unresolved confusion, unresolved frustration, pleasant frustration, and energizing frustration. It would open up a place to consider new types of frustration and confusion, with immediate implications for where we should look—is there such a thing as pleasant confusion or energizing confusion? The existence and commercial success of television shows with the seeming primary goal of confusing their viewers certainly argues for such a construct. As shown in Fig. 2, the different forms of confusion and frustration can also be mapped to locations within the Circumplex model, placed according to approximate relationships to emotional valence and arousal. Representing confusion and frustration within this constellation would also provide a framework for understanding new manifestations of confusion and frustration, and relating them to each other. It would acknowledge that some of the manifestations of confusion and frustration have more in common with each other (e.g., unresolved confusion and unresolved frustration; resolved confusion and resolved frustration; extended confusion and extended frustration; brief confusion and brief frustration) than with other manifestations within their own category, and provide a basis for explaining these commonalities and differences.

**Fig. 3:**
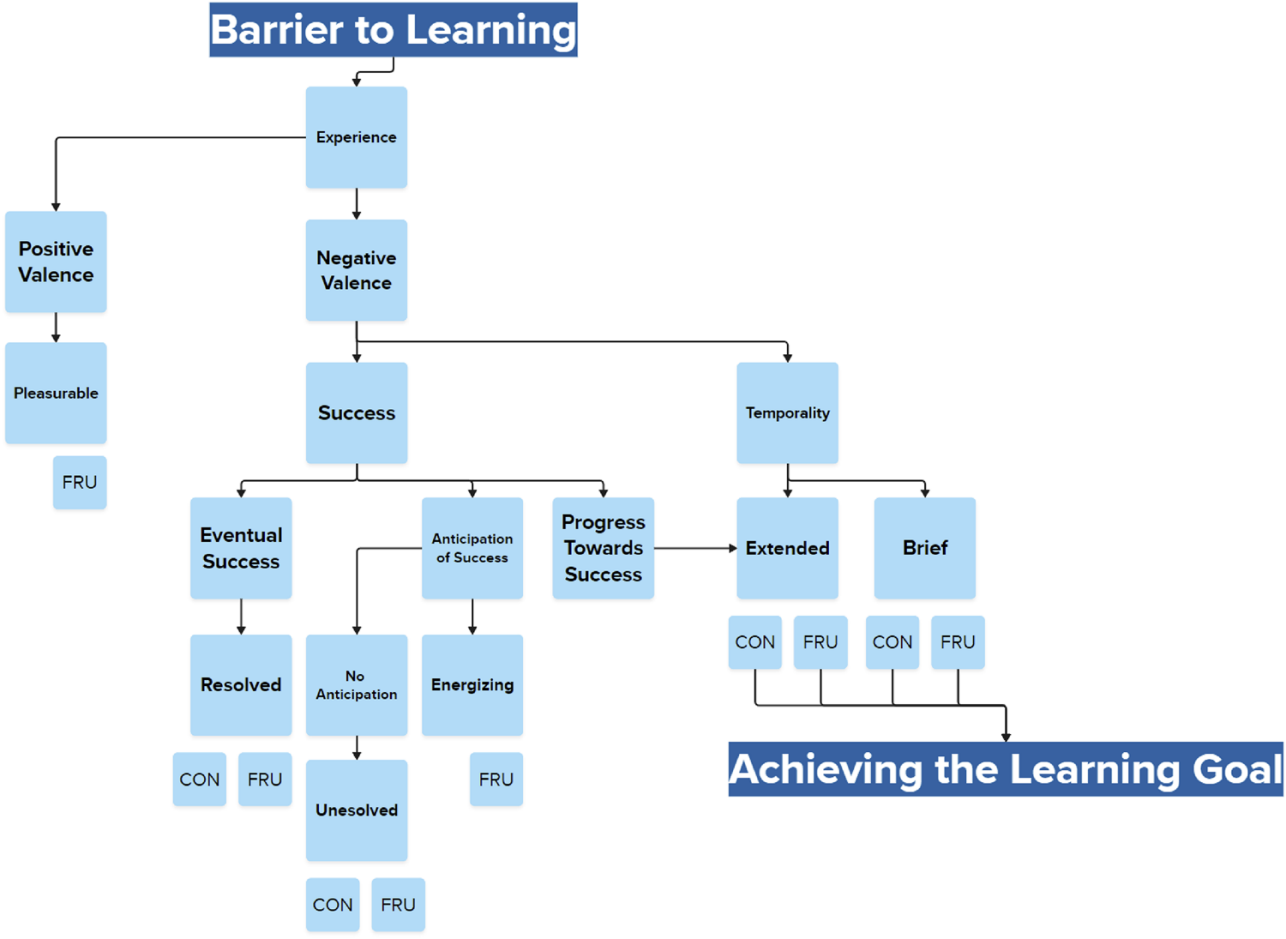
Factors contributing to various forms of confusion and frustration.

A representation of confusion and frustration's varied manifestations as a constellation would go beyond the two dimensions of Circumplex to become multidimensional, considering factors such as temporality, valence, arousal, eventual success, progress toward success, and the anticipation of success. It could be expressed in diagrams similar to the OCC model of affect (Ortony et al., 1988) or a multidimensional set of two‐dimensional diagrams. For example, as Fig. 3 shows, the various factors producing different forms of confusion and frustration could be represented in an OCC‐inspired diagram focusing on a narrower range of affect (and factors influencing affect) than OCC captures. Understanding the relationship between these dimensions would then become a core goal of future research on confusion and frustration. Researchers could ask questions like: Is temporality simply a function of success‐related dimensions? How does duration impact arousal and valence, and what are the conditions that influence this development? And how do metacognitive awareness (the ability to accurately detect an affective state occurring) and cognition impact transitions within the multidimensional space of confrustion? For example, if a learner views confusion as a natural part of the learning process, are they relatively less likely to move from unresolved confusion to unresolved frustration, or perhaps even likely to move from unresolved confusion to pleasant frustration?

Overall, given the motivating properties of frustration discussed in Amsel (1994), overlapping findings in prior studies (e.g., Bosch & D'Mello, 2014; Bosch & D'Mello, 2017; Botelho et al., 2018; Liu et al., 2013), but inconsistent empirical findings regarding the impact of confrustion on learning outcomes (e.g., Mogessie et al., 2020; Richey et al., 2019; Richey et al., 2021; Richey et al., 2019), there may be value for future research studies to reconceptualize confrustion as a multifaceted and interconnected emotional experience that cannot be reduced to the sum of its parts (e.g., confusion or frustration).

### 3.2 Confrustion and learning

If we reframe our thinking on confusion and frustration as a confrustion constellation, then perhaps this provides a new angle on a question that has—well—confused and frustrated many researchers over the last decade: what is the relationship between confusion/frustration and learning? As noted above, the answer to this question in aggregate across studies has been “it's unclear.” But if we instead ask what the relationship is between confrustion and learning, we no longer expect a single answer. Instead, the question rapidly becomes one of asking, what aspects of the experiences of confrustion are associated with differences in learning? And in turn, this calls for research that simultaneously varies the dimensions of confrustion, and investigates their association with learning.

One could imagine, for instance, a program of research where learners are presented with a situation where they genuinely want to understand a topic but there are one or more impasses or barriers preventing that (perhaps a challenging but fun game; perhaps a topic known to be difficult within their course of study). They could then be presented with a variety of materials and supports that systematically vary the comprehensibility of material, the presence and seriousness of contradictions, the delay in providing explanatory supports, the timing for how long to wait when a student has left the confrustion constellation (i.e., is no longer confused or frustrated) before returning them to it, the presence of gamified rewards of various types when they reach comprehension milestones (and the role of the reward environment in general), the apparent career and contextual relevance of the material, and so on. Retrospective emote‐aloud protocols (D'Mello et al., 2006) could be used to verify and better understand the affective experience under these conditions, and post‐tests (or even mid‐tests) could be used to verify learning, as well as the judgment of learning protocols.

A similar program of research could be carried out, somewhat less efficiently, across a span of natural learning activities—say, in a year‐long curriculum, or even in a future research synthesis across a number of unrelated studies that less systematically vary these variables. Ultimately, by considering these variables, though, it will be possible to understand the role that the varied manifestations of confrustion play in learning. It would also become possible to better understand and predict student trajectories through (and in and out of) the confrustion space (cf. Botelho et al., 2018; D'Mello & Graesser, 2012), and what factors influence those trajectories.

As we develop this theoretical understanding, it would become possible to identify the development of different forms of confrustion in real time (in a richer fashion than current automated detectors of confusion and frustration), and to know which interventions and supports would help move students through that space in ways more beneficial to their learning and ultimate engagement with the topic. By understanding confusion and frustration as part of the same constellation, we can not only acknowledge their underlying and interconnected properties of motivation and goals toward informational processing, comprehension, and learning, but we can also adopt a more complex understanding of a learner's epistemic emotional experience to better inform pedagogical practices and interventions.

### 3.3 Studying confrustion: Toward new elicitation methods

In developing a theoretical understanding of confrustion, a multimodal and mixed‐methods approach may be necessary. As discussed above, past models have made relatively simple assumptions about the physiological and behavioral associations of confusion and frustration by considering each of them in a unitary fashion. Work adhering to this paradigm has made the assumption that confusion and frustration are separate emotional states (D'Mello & Graesser, 2014; D'Mello & Graesser, 2012). As a result, this work often attempts to *detect* a single form of each confusion or frustration using physiological and behavioral indicators (log files, facial expressions, galvanic skin response, posture sensing, eye tracking), searching for distinct manifestations of each (e.g., what combination of physiological and behavioral data are valid and indicative of its presence or absence) (AlZoubi et al., 2012; Botelho et al., 2017; Cloude et al., 2022; Dever et al., 2022; Jiang et al., 2018; Kapoor et al., 2007; McDaniel et al., 2007).

However, this paradigm oversimplifies the underlying complexity and interconnected nature of confusion and frustration. We argue that confusion and frustration share a key commonality as they share the same underlying motivation—the desire to learn or understand new information, and some level of challenge in doing so. This shared motivational foundation suggests that confusion and frustration are not *entirely* separate emotions but rather interconnected responses to the challenge of overcoming barriers to learning (see Fig. 2 above). For example, mild confusion when unresolved could escalate into frustration; that frustration might, in turn, exacerbate confusion (e.g., frustration clouding cognitive processing). This interconnectedness could be represented as cyclical, rather than separate and linear, as suggested by D'Mello and Graesser (2012).

An alternate approach for *understanding* confrustion would, instead, ask what do different data channels and their modalities tell us, in finer‐grained detail, about the learner's momentary experiences. For example, how does arousal (measured by galvanic skin response) track (or follow, or precede) changes in a student's affective experience? These data may offer a means to detect if and when a learner has increased or decreased in arousal over time as they move through the confrustion constellation.

Analyzing this also depends on better labeling where a student is within the confrustion constellation. As such, it becomes necessary to replace existing methods of obtaining labels of a student's experience—currently usually obtained through labeling confusion, frustration, or other discrete affective states through more or less expert field observations (Ocumpaugh et al., 2015) or simple self‐report (Pekrun et al., 2017; Sabourin et al., 2011)—with more complex measures. For example, one such approach would be to conduct more in‐depth retrospective emote‐alouds. In such a method, the learner could be prompted to first take a pass where they watch through their own past interaction and simply state high‐level affect labels, much as in existing methods for retrospective emote‐aloud (D'Mello et al., 2006). After that, however, the learner could be asked to re‐review the cases where they reported confusion or frustration, to provide deeper detail on what they were feeling, either in their own words, or with reference to a set of terms related to deeper positioning on the confrustion constellation. Better labels could also be obtained by in‐depth in‐the‐moment interviews that would probe the student's cognition and affect in greater detail. In recent years, researchers have used automated detectors of confusion and frustration to select students for in‐the‐moment interviews that can occur under a minute after the emotion is detected (Baker et al., 2021, Baker et al., 2024). During these interviews, the student could be prompted to discuss their affective experience in greater detail and depth, perhaps being asked to respond about multiple dimensions relevant to the confrustion constellation, or being shown a diagram/diagrams of the confrustion constellation and asked to point to where their recent emotion is. Finally, existing in‐the‐moment pop‐up surveys could start as they are currently, giving a list of affective states and emoticons to select from, but if the student selected confusion or frustration, they could then be given a list of sliders for different dimensions of the confrustion constellation or a diagram of the constellation to click on. These approaches could help researchers develop a richer understanding of student experience.

However, these approaches would by nature be more disruptive than existing measurements, so ideally they would only be deployed in specific circumstances, such as when a specific combination of student experience is encountered. Researchers could use automated detection of confrustion, in combination with other data (e.g., on‐site observations or physiology), to identify situations where a deeper understanding of student cognition and emotion is needed and prompt the necessary data collection. Measuring multiple dimensions of confrustion when it occurs could also be achieved by collecting data on confrustion‐relevant behaviors alongside expressions, physiological measurements, cognitive measurements, and even neurological measurement, in addition to self‐report of various forms. In order to achieve this, future researchers may need to utilize interaction‐dominant methods (as opposed to only using single component‐dominant methods), which quantify the degree of interactions among multiple signals which may help define different subsets of confrustion (e.g., investigating which facial expressions co‐occur with specific confused or frustrated behaviors and how these interactions are differentially associated with learning [Cloude et al., 2024]). Combining different forms of data may shed light on if and where a learner's experience falls within the confrustion constellation, allowing a more nuanced representation of the different forms of confrustion to study its effect on learning and other phenomena. In doing this, it might be possible to rely at first upon the current oversimplified detection of confusion and frustration, and then to use the insights gained to produce more sophisticated models and understanding. Again, an early example of this approach is seen in work that uses automated detectors of frustration to select students for classroom interviews and notify classroom‐embedded researchers in real time (Baker et al., 2021).

### 3.4 The confrustion constellation: Benefits of a new framework

Considering confrustion as a constellation clearly creates several new methodological challenges. At the same time, it presents several opportunities that the existing ways of conceptualizing these affective states fail to provide. First of all, there are several phenomena that existing models fail to capture, particularly the commonalities between these affective states’ origins, their most common manifestations, and the outcomes associated with similar manifestations, such as extended confusion and extended frustration. Moreover, existing models do not adequately account for the diversity in these two forms of affect, and do not explain the inconsistencies and contradictions in past research on affect dynamics. By explicitly acknowledging and then studying the multidimensionality of confusion and frustration, we can better understand the dimensions that impact these affective states’ manifestations and effects. In doing so, we can obtain the evidence needed to turn the tentative models shown in Figs. 2 and 3 into crystallized, well‐documented theoretical models that provide descriptive insights and better predictive opportunities than what is possible with current models of confusion and frustration.

This new approach leads to the question of what dimensions are most important to the manifestation of confusion and frustration, and how they combine with the antecedents or triggers of confusion and frustration to produce specific affective manifestations (see Fig. 3). It encourages us to explore uncharted aspects of this constellation, such as why pleasant confusion has not been reported when pleasant frustration is at least a somewhat known quantity. It also supports us in exploring the transition between different aspects of confrustion as opposed to simply confusion and frustration as unitary states. For example, unresolved confusion may lead to extended frustration, but a learner's metacognitive awareness of their state might prevent this transition or even lead to a positive affect. Similarly, the experience of pleasant frustration may serve as an antecedent to resolved frustration. It is even possible that reconceptualizing (and measuring) confusion and frustration on these more complex terms may resolve the contradictions and lack of replicability in past studies of affect dynamics (Karumbaiah et al., 2023).

By adopting this constellation model, we open up possibilities for new research methodologies and pedagogical interventions that can respond to the diverse emotional experiences of learners in more sensitive ways. Overall, one clear implication of this new model is that it leads to reconceptualizing emotional states as multivariate and thinking about them and measuring them in ways that capture more of their rich complexity. Rather than treating confusion and frustration as distinct and separate states, this model encourages us to investigate how different dimensions—such as duration, arousal, and progress toward success—interact to influence learning experiences and outcomes. Understanding these interactions will not only lead to richer models of affect but also help educators and researchers design targeted interventions that guide students through the confrustion space in a way that maximizes learning.

## 4 Conclusion

There have been decades of work attempting to understand frustration and confusion, with particularly strong interest in the last 15 years. Overall, this work has yielded considerable understanding of these affective states, but it has also generated a number of findings that simultaneously suggest that these labels fail to capture both the complexity and heterogeneity of each of these categories, while ignoring key links and similarities between confusion and frustration that cannot be reduced to simple formulations such as “unresolved confusion becomes frustration” (one of the links discussed in D'Mello & Graesser, 2012). Within this paper, we review the history of research on confusion and frustration, considering both theoretical models and empirical findings. Our review demonstrates the complexity of affect in this space and the sheer quantity of phenomena that a complete model of these aspects of affect must account for—from the interrelationships between how duration impacts the experience of both confusion and frustration, to the phenomenon of pleasurable frustration, an experience that seemingly contradicts the very definition of frustration used in many accounts.

Within these pages, we propose an alternate theoretical paradigm for these affective states: a confrustion constellation, where confusion and frustration manifest in a number of ways but there are meaningful commonalities among all of the manifestations. This paradigm builds on the commonalities that many of the manifestations of frustration and confusion share, that when either is varied along specific dimensions they seem to vary together (e.g., extended confusion and frustration; brief confusion and frustration; unresolved confusion and frustration; resolved confusion and frustration), and that the central manifestations of these two affective states seem more similar than either state's outlier manifestations (i.e., pleasant frustration). We argue that studying confusion and frustration in this fashion requires different empirical strategies (across studies) and a different combination of methods, in order to develop a full understanding of confusion and frustration that beneficially impacts learning design. In particular, studying confusion and frustration in this fashion helps us move beyond trying to resolve contradictions in the empirical literature to instead focus on better understanding the role that multiple aspects of context play in how confrustion manifests and impacts on learning.

In developing a comprehensive account of confrustion, a great number of factors must be considered. One that we have not treated in detail—but may be extremely relevant—is that of identity, language, and culture. Confusion and frustration are labeled in different ways in different languages and cultures—there is not an obvious and simple translation for one or both of these terms into several languages. In addition, in work supporting the deployment of classroom observation methods in several countries (Baker, Ocumpaugh, & Andres, 2020), local partner researchers have stated that frustration does not occur in their students and that students instead experience contempt for the content, a different cognitive reframing of the same experience. This type of linguistic and cognitive difference is likely to influence how the confrustion constellation manifests in different linguistic, social, and cultural contexts, particularly around how the student may appraise feelings of conflict as well as their willingness to disclose these externally (and recognize them internally). The relationships between confrustion and learning also appear to vary between different countries, although other contextual factors were not fully accounted for between studies (see review in Karumbaiah et al., 2022). For this reason, researchers are urged to utilize some affect detection methods with caution, such as facial tracking systems, which often define the presence of confusion or frustration by training algorithms on data collected from white, English‐speaking male faces that originate from western cultures. Ecological validity is also open to question since many of these systems were trained on corpuses where participants were asked to express confusion and frustration in a controlled laboratory setting.

Finally, differences in student background beyond culture and language may influence the cognition that drives confrustion. A student with personal experience of poverty and/or discrimination may interpret reward, the potential for reward, the potential for success, and the relevance of an experience differently than a student who has not had these personal experiences. Such factors must be taken into account in a full and comprehensive model of these phenomena.

By proposing that confusion and frustration exist as several points in a closely interrelated space, the confrustion constellation challenges existing categorical theories, suggesting that confusion and frustration are fluid, interconnected, and multidimensional. Adopting a multidimensional approach which assumes that confusion and frustration are not neatly separated but are deeply linked allows for a more accurate representation of how learners feel and the processes surrounding these feelings.

Overall, we believe that our field's confusion and frustration about confusion and frustration can be resolved. The duration of our confusion and frustration has been lengthy, but switching our framing of the experience can help us to find new avenues for understanding. We can get past our cognitive disequilibrium and dissonance to a new equilibrium of comprehension. And when we resolve this confusion and frustration, we will better understand how to leverage confrustion to help optimize learning and learning experiences. The implications of adopting a multidimensional model of confrustion could lead to developing new methodological and analytical techniques for measuring and modeling emotional states in deeper complexity rather than as fixed, separate categories. This shift in models would thus enable more precise and real‐time assessments of emotions, creating opportunities for just‐in‐time pedagogical interventions in both research and practice.

## Ethics statement

The theoretical work presented here does not involve human subjects research or data and, therefore, did not require ethics approval at our universities. No known conflicts of interest were involved. All figures have been created by our team and we grant permission for their use.
